# Complete genome sequence of *Dyadobacter fermentans* type strain (NS114^T^)

**DOI:** 10.4056/sigs.19262

**Published:** 2009-09-24

**Authors:** Elke Lang, Alla Lapidus, Olga Chertkov, Thomas Brettin, John C. Detter, Cliff Han, Alex Copeland, Tijana Glavina Del Rio, Matt Nolan, Feng Chen, Susan Lucas, Hope Tice, Jan-Fang Cheng, Miriam Land, Loren Hauser, Yun-Juan Chang, Cynthia D. Jeffries, Marcus Kopitz, David Bruce, Lynne Goodwin, Sam Pitluck, Galina Ovchinnikova, Amrita Pati, Natalia Ivanova, Konstantinos Mavrommatis, Amy Chen, Krishna Palaniappan, Patrick Chain, Jim Bristow, Jonathan A. Eisen, Victor Markowitz, Philip Hugenholtz, Markus Göker, Manfred Rohde, Nikos C. Kyrpides, Hans-Peter Klenk

**Affiliations:** 1 DSMZ - German Collection of Microorganisms and Cell Cultures GmbH, Braunschweig, Germany; 2 DOE Joint Genome Institute, Walnut Creek, California, USA; 3 Los Alamos National Laboratory, Bioscience Division, Los Alamos, New Mexico, USA; 4 Oak Ridge National Laboratory, Oak Ridge, Tennessee, USA; 5 Biological Data Management and Technology Center, Lawrence Berkeley National Laboratory, Berkeley, California, USA; 6 Lawrence Livermore National Laboratory, Livermore, California, USA; 7 University of California Davis Genome Center, Davis, California, USA; 8 HZI - Helmholtz Centre for Infection Research, Braunschweig, Germany

**Keywords:** mesophile, free-living, non-pathogenic, aerobic, chains of rods, *Cytophagaceae*

## Abstract

*Dyadobacter fermentans* (Chelius and Triplett, 2000) is the type species of the genus *Dyadobacter*. It is of phylogenetic interest because of its location in the *Cytophagaceae,* a very diverse family within the order ‘*Sphingobacteriales’. D. fermentans* has a mainly respiratory metabolism, stains Gram-negative, is non-motile and oxidase and catalase positive. It is characterized by the production of cell filaments in aging cultures, a flexirubin-like pigment and its ability to ferment glucose, which is almost unique in the aerobically living members of this taxonomically difficult family. Here we describe the features of this organism, together with the complete genome sequence, and its annotation. This is the first complete genome sequence of the sphingobacterial genus *Dyadobacter*, and this 6,967,790 bp long single replicon genome with its 5804 protein-coding and 50 RNA genes is part of the *** G****enomic* *** E****ncyclopedia of* *** B****acteria and* *** A****rchaea * project.

## Introduction

**Figure 1:**
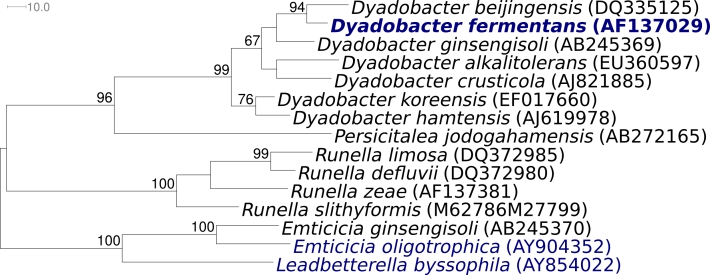
Phylogenetic tree of *D. fermentans* strain NS114^T^, all type strains of the genus *Dyadobacter* and the most closely related *Cytophagaceae* type strains, inferred from 1,378 aligned characters [2,3] of the 16S rRNA sequence under the maximum parsimony criterion [4]. The tree was rooted with *Emticicia* and *Leadbetterella*, members of the family *Cytophagaceae*. The branches are scaled in terms of the minimal number of substitutions across all sites. Numbers above branches are support values from 1,000 bootstrap replicates if larger than 60%. Strains with a genome sequencing project registered in GOLD [5] are printed in blue; published genomes in bold.

Strain NS114^T^ (= DSM 18053 = ATCC 700827 = CIP 107007) is the type strain of *Dyadobacter fermentans*, which is the type species of the genus *Dyadobacter. D. fermentans* was described by Chelius and Triplett in 2000 [1] as mainly aerobic, but also able to grow by fermentation of glucose, Gram negative and nonmotile. The organism is of significant interest for its position in the tree of life, because the genus *Dyadobacter* (currently seven species, Figure 1) is rather isolated within the family *Cytophagaceae* [6], as it has less than 88% similarity of the 16S rRNA gene sequence to any other bacteria with standing in nomenclature, with *Persicitalea* and *Runella* as closest neighboring genera. Here we present a summary classification and a set of features for *D. fermentans,* strain NS114^T^ (Table 1), together with the description of the complete genomic sequencing and annotation.

**Table 1:** Classification and general features of *D. fermentans* NS114^T^ based on the MIGS recommendations [7]

**MIGS ID**	**Property**	**Term**	**Evidence code**
	Current classification	Domain *Bacteria*	TAS [6]
		Phylum ’Bacteroidetes’	TAS [6]
		Class ’*Sphingobacteria’*	TAS [6]
		Order ’*Sphingobacteriales’*	TAS [6]
		Family *Cytophagaceae*	TAS [8]
		Genus *Dyadobacter*	TAS [1]
		Species *Dyadobacter fermentans*	TAS [1]
		Type strain NS114	
	Gram stain	negative	TAS [1]
	Cell shape	rods in pairs or chains	TAS [1]
	Motility	nonmotile	TAS [1]
	Sporulation	non-sporulating	TAS [1]
	Temperature range	mesophilic	TAS [1]
	Optimum temperature	not reported	TAS [1]
	Salinity	tolerates up to 15g NaCl/L	TAS [1]
MIGS-22	Oxygen requirement	essentially aerobic	
	Carbon source	carbohydrates such as sugars, glucose and sucrose, sugar alcohols and carbonic acids but no polymers such as starch, cellulose or gelatin	TAS [1]
	Energy source	glucose and sucrose by fermentation aerobically, no acid production from glucose detectable	TAS [1]
MIGS-6	Habitat	endophytic in stems of maize plants	TAS [1] TAS [12] TAS [9]
		cysts of nematode *Heterodera glycines*	
		contaminated soil	
MIGS-15	Biotic relationship	free-living	
MIGS-14	Pathogenicity	none	TAS [1]
	Biosafety level	1	TAS [10]
	Isolation	surface sterilized stems of maize plants grown in sterile soil without nitrogen fertilizer under green house conditions	TAS [1]
MIGS-4	Geographic location	Madison-Wisconsin; USA	NAS
MIGS-5	Sample collection time	not reported	
MIGS-4.1 MIGS-4.2	Latitude, Longitude	43.1, 89.4	NAS
MIGS-4.3	Depth	Not reported	
MIGS-4.4	Altitude	Not reported	

Evidence codes - IDA: Inferred from Direct Assay (first time in publication); TAS: Traceable Author Statement (i.e., a direct report exists in the literature); NAS: Non-traceable Author Statement (i.e., not directly observed for the living, isolated sample, but based on a generally accepted property for the species, or anecdotal evidence). These evidence codes are from the Gene Ontology project [11]. If the evidence code is IDA the property was directly observed for a live isolate by one of the authors or an expert mentioned in the acknowledgements.

### Classification and features

Strain NS114^T^ was isolated in a study on the endophytic community of maize plants, where bacteria were isolated from plants which had been grown using surface sterilized seeds in autoclaved synthetic soil in greenhouses [1]. Plant stems were surface sterilized and crushed prior to plating. The organism was found in stem tissues of plants which were cultivated on a nitrogen-free nutrient solution, but not in the nitrogen-fertilized counterparts. The type strain of *Runella zeae* (NS12^T^) was co-isolated from the same material, although no microscopic evidence has been presented to date that members of these species were living within the plant tissue [1].

Members of the species *D. fermentans* were regularly found in cysts of the soybean nematode *Heterodera glycines* [12]. *D. fermentans* was also isolated from contaminated soil as a result of its ability to grow on 7,8-benzoquinoline [9], a nitrogen-containing heterocyclic aromatic hydrocarbon (azaarene) widely distributed in products of incomplete combustion processes, with toxic and cancerogenic effects. Two isolates from a lime stone cavern in Arizona share significant 16S rRNA gene sequence similarity of 98% (DQ207364 and DQ207362). Only a few closely related phylotypes from environmental samples and global surveys are recorded to be highly related to *D. fermentans*, a fact suggesting that members of this taxon are not very abundant: three clones of uncultured bacteria of a deep sea sediment collected at the Western Tropical Pacific Warm Pool in the Pacific Ocean (AM085468, AM085473 and AM085489) and one of the activated sludge of a membrane bioreactor (EU283373) are recorded (as of May 2009). Intrageneric similarity within the genus *Dyadobacter* is rather low [13].

**Figure 2:**
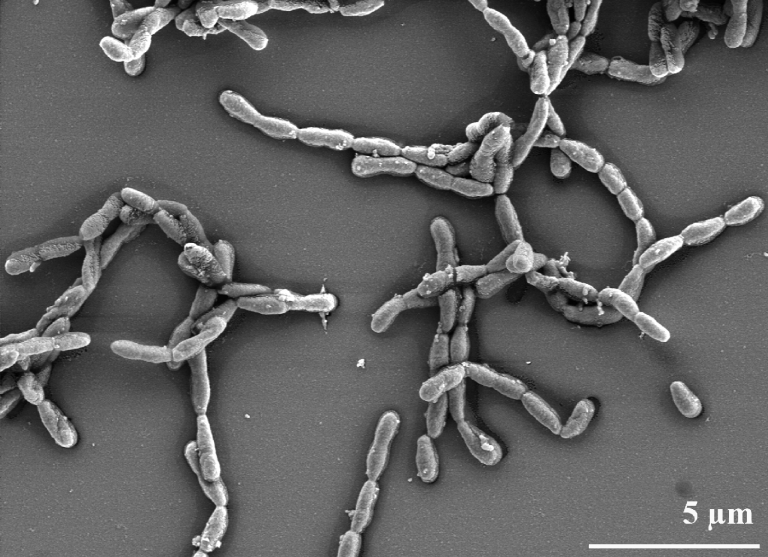
Scanning electron micrograph of *D. fermentans* NS114^T^

*D. fermentans* has been recognized by its flexirubin-like yellow pigment and by its growth as flocculent filaments of ovoid rods in old cultures [1]. The rod-shaped cells of strain NS114^T^ occur polarly attached in groups of 2-4 cells during logarithmic growth, cells being frequently arranged at an angle to give V-formations. As the culture ages, irregular filaments or ovoid rods form (Figure 2) which sediment as fluffs in liquid culture [1]. The cells are Gram negative, non-motile and non-sporulating, oxidase and catalase positive. Like many plant associated bacteria, strain NS114^T^ produces copious amounts of slime when grown on nitrogen-limited agar. The organism grows aerobically, however it is also reported to be able to ferment glucose and sucrose in the O/F-test in Hugh-Leifson medium [1,13], which according to our own observations might be an experimental artifact due to prolonged incubation. The colony color of strain NS114^T^ is yellow to orange [1,13]. The absorbance maximum of an ethanol extract of the cells is at 450 nm, and the absorbance peak broadens under alkaline conditions, which is a typical feature of flexirubin-like pigments [1]. This pigment was found in all six species of the genus *Dyadobacter* thus confirming that the presence of the pigment is a property of the genus [14-18].

Figure 1 shows the phylogenetic neighborhood of *D. fermentans* strain NS114^T^ in a 16S rRNA based tree. Analysis of the four 16S rRNA gene sequences in the genome of strain NS114^T^ indicated that three copies are almost identical, and one differs by seven nucleotides from these. The sequences of the three identical 16S rRNA genes differ by two nucleotides from the previously published 16S rRNA sequence generated from DSM 18053 (AF137029). The slight differences between the genome data and the reported 16S rRNA gene sequence are probably the result of sequencing errors in the previously reported sequence data.

The whole cell fatty acid pattern of strain NS114^T^ is dominated by unsaturated, and saturated *iso*-branched, straight chain unsaturated and large amounts of *iso*-branched, hydroxylated species. Major components are iso-C_15:0_-2OH and/or C_16:1_ω7c (43.5%), C_16:1_ ω5c (17.5%) and *iso*-C_15:0_ (16.8). Considerable amounts of the 3-hydroxylated fatty acids *iso*-C_15:0_-3OH_,_ C_16:0_-3OH and *iso*-C_17:0_ -3OH are detected [1]. The quinone composition has not been investigated for strain NS114^T^. The main component reported for the closely related type strains of *D. ginsengisoli* and *D. alkalitolerans* is menaquinone MK-7 [16,18]. Cells of strain NS114^T^ contain spermidine as the major cellular polyamine and putrescine, cadaverine and spermine as minor components. The latter compound was not detected in any of the three other strains studied to date of the family *Cytophagaceae*, representing *Flexibacter flexilis, Microscilla marina,* and *D. beijingensis* [19]. The polar lipid composition has not been investigated in either this strain or other members of the genus *Dyadobacter*.

## Genome sequencing and annotation

### Genome project history

This organism was selected for sequencing on the basis of its phylogenetic position, and is part of the *** G****enomic* *** E****ncyclopedia of* *** B****acteria and* *** A****rchaea * project. The genome project is deposited in the Genomes OnLine Database [5] and the complete genome sequence in GenBank. Sequencing, finishing and annotation were performed by the DOE Joint Genome Institute (JGI). A summary of the project information is shown in Table 2.

**Table 2:** Genome sequencing project information

**MIGS ID**	**Property**	**Term**
MIGS-31	Finishing quality	Finished
MIGS-28	Libraries used	Two genomic Sanger libraries: 8 kb pMCL200 and fosmid pcc1Fos
MIGS-29	Sequencing platforms	ABI3730
MIGS-31.2	Sequencing coverage	10.6× Sanger
MIGS-30	Assemblers	Phred/Phrap/Consed
MIGS-32	Gene calling method	Prodigal, GenePrimp
	INSDC / Genbank ID	CP001619
	Genbank Date of Release	July 31, 2009
	GOLD ID	Gc01069
	Database: IMG-GEBA	2501416930
	NCBI project ID	20829
MIGS-13	Source material identifier	DSM 18053
	Project relevance	Tree of Life, GEBA

### Growth conditions and DNA isolation

*D. fermentans* NS114^T^, DSM18053, was grown in DSMZ medium 830 (R2A Medium) at 28°C [20]. DNA was isolated from 1-1.5 g of cell paste using Qiagen Genomic 500 DNA Kit (Qiagen, Hilden, Germany) with a modified protocol (FT) for cell lysis as described in Wu *et al.* [21].

### Genome sequencing and assembly

The genome was sequenced using a combination of 8 kb and fosmid DNA libraries. All general aspects of library construction and sequencing performed at the JGI can be found at the JGI website (http://www.jgi.doe.gov). Draft assemblies were based on 86,260 total reads. The Phred/Phrap/Consed software package (http://www.phrap.com) was used for sequence assembly and quality assessment [22-24]. After the shotgun stage, reads were assembled with parallel phrap. Possible mis-assemblies were corrected with Dupfinisher or transposon bombing of bridging clones [25]. Gaps between contigs were closed by editing in Consed, custom primer walk or PCR amplification. A total of 1,042 additional reactions were necessary to close gaps and to raise the quality of the finished sequence. The error rate of the completed genome sequence is less than 1 in 100,000. Together all libraries provided 10.6x coverage of the genome.

### Genome annotation

Genes were identified using Prodigal [26] as part of the Oak Ridge National Laboratory genome annotation pipeline , followed by a round of manual curation using the JGI GenePRIMP pipeline [27]. The predicted CDSs were translated and used to search the National Center for Biotechnology Information (NCBI) nonredundant database, UniProt, TIGRFam, Pfam, PRIAM, KEGG, COG, and InterPro databases. Additional gene prediction analysis and manual functional annotation was performed within the Integrated Microbial Genomes Expert Review (IMG-ER) platform [28].

### Genome properties

**Figure 3:**
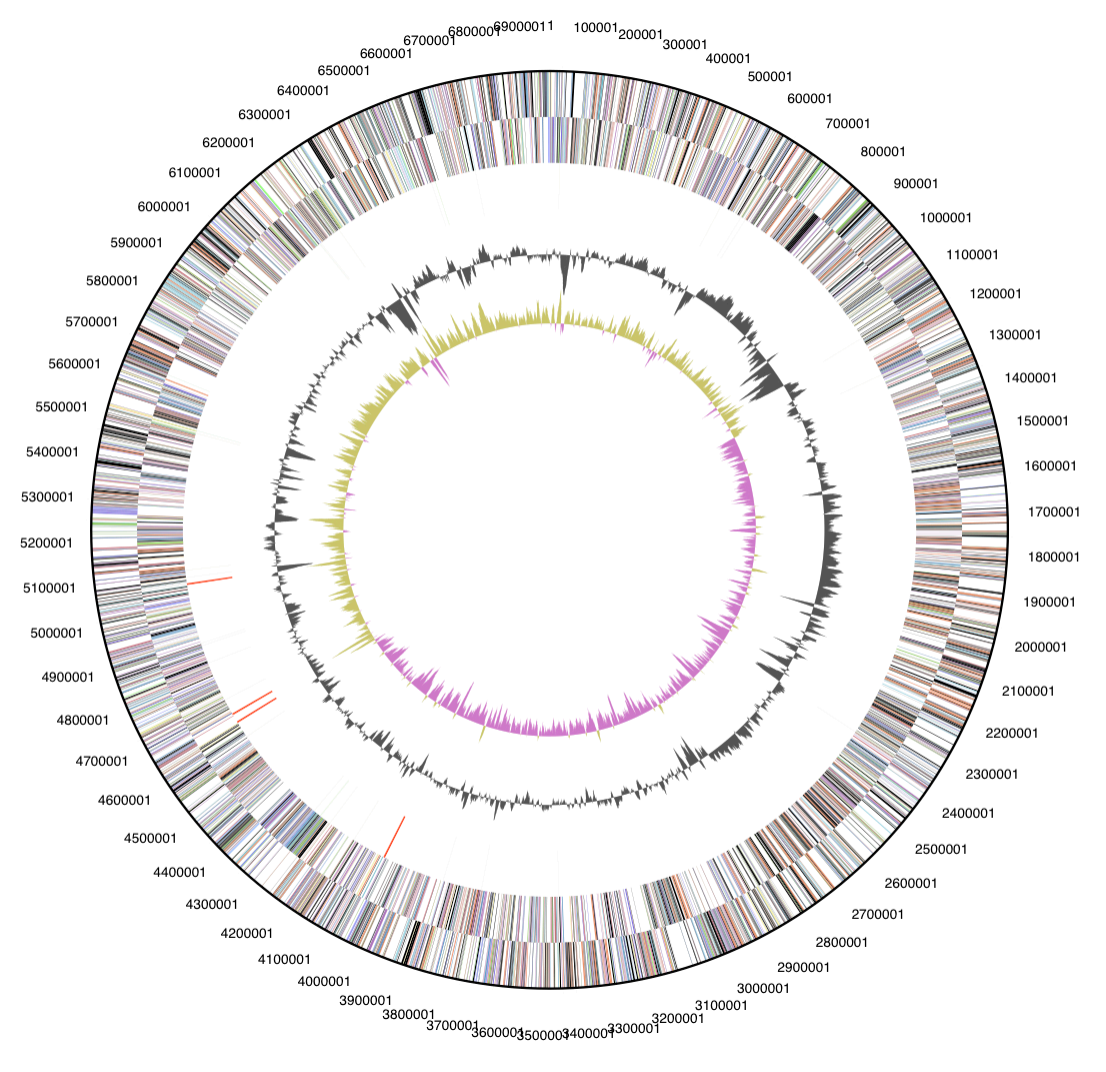
Graphical circular map of the genome. From outside to the center: Genes on forward strand (color by COG categories), Genes on reverse strand (color by COG categories), RNA genes (tRNAs green, rRNAs red, other RNAs black), GC content, GC skew.

The genome is 6,967,790 bp long and comprises one main circular chromosome with a 51.4% GC content (Table 3, Figure 3). Of the 5,854 genes predicted, 5,804 were protein coding genes, and 50 were RNAs. In addition, 85 pseudogenes were identified. The majority of the protein-coding genes (64.7%) were assigned with a putative function while those remaining were annotated as hypothetical proteins. The properties and the statistics of the genome are summarized in Table 3. The distribution of genes into COG functional categories is presented in Table 4.

**Table 3:** Genome Statistics

**Attribute**	Value	% of Total	
Genome size (bp)	6,967,790	100.00%
DNA Coding region (bp)	6,343,890	91.05%
DNA G+C content (bp)	3,591,547	51.54%
Number of replicons	1	
Extrachromosomal elements	0	
Total genes	5,854	100.00%
RNA genes	50	0.85%
rRNA operons	4	
Protein-coding genes	5,804	99.15%
Pseudo genes	85	1.45%
Genes with function prediction	3,790	64.74%
Genes in paralog clusters	1,250	21.35%
Genes assigned to COGs	3,702	63.24%
Genes assigned Pfam domains	3,853	65.82%
Genes with signal peptides	1,760	30.06%
Genes with transmembrane helices	1,239	21.17%
CRISPR repeats	0	

**Table 4:** Number of genes associated with the general COG functional categories

Code	Value	% of total	Description
J	171	2.9	Translation
A	0	0.0	RNA processing and modification
K	392	6.8	Transcription
L	157	2.7	Replication, recombination and repair
B	0	0.0	Chromatin structure and dynamics
D	23	0.4	Cell cycle control, mitosis and meiosis
Y	0	0.0	Nuclear structure
V	111	1.9	Defense mechanisms
T	343	5.9	Signal transduction mechanisms
M	345	5.9	Cell wall/membrane biogenesis
N	13	0.2	Cell motility
Z	1	0.0	Cytoskeleton
W	0	0.0	Extracellular structures
U	56	1.0	Intracellular trafficking and secretion
O	110	1.9	Posttranslational modification, protein turnover, chaperones
C	179	3.1	Energy production and conversion
G	335	5.8	Carbohydrate transport and metabolism
E	247	4.4	Amino acid transport and metabolism
F	75	1.3	Nucleotide transport and metabolism
H	190	3.3	Coenzyme transport and metabolism
I	143	2.5	Lipid transport and metabolism
P	295	5.1	Inorganic ion transport and metabolism
Q	102	1.8	Secondary metabolites biosynthesis, transport and catabolism
R	540	9.3	General function prediction only
S	345	5.9	Function unknown
-	2102	36.2	Not in COGs
